
JOURNAL INFORMATION
==============================
NLM Title Abbreviation: ScientificWorldJournal
Journal ID: ScientificWorldJournal
NLM Journal ID: 101131163
Journal ID: TSWJ

The Scientific World Journal

ISSN: 2356-6140
EISSN: 1537-744X
Publisher: Wiley

ARTICLE INFORMATION
==============================
PMCID: PMC6009606
PMID: 29973823
DOI: 10.1100/tsw.2002.49
Article ID: 925636
Article version: 1
Subjects: Short Report

Detection of Membrane Protein Interactions Using Split Ubiquitin-Based Membrane Yeast Two-Hybrid Technology

Thaminy Safia 1
Auerbach Daniel 2
Galeuchet-Schenk Barbara 2
Scheper Wiep 3
Ramisch Karin 3
Stagljar Igor 1 2 *
Institute of Veterinary Biochemistry and Molecular Biology, University of Zurich, Switzerland
Dualsystems Biotech Inc, Zurich, Switzerland
University of Cambridge, CIMR, UK
*Igor Stagljar: stagljar@vetbio.unizh.ch, stagljar@dualsystems.com
Publication date: 2002
Electronic publication date: 2002 May 29
Volume: 2
First page: 104
Last page: 105
Copyright: Copyright © 2002 Safia Thaminy et al.
Copyright year: 2002
License: This is an open access article distributed under the Creative Commons Attribution License, which permits unrestricted use, distribution, and reproduction in any medium, provided the original work is properly cited.
License URL: https://creativecommons.org/licenses/by/3.0/

Funding: Bonizzi-Theler Stiftung Zürcher Krebsliga Walter Honegger Stiftung Swiss National Science Foundation 31-58798.99

==============================
Advances in Gene Technology: The Genome and Beyond –
Structural Biology for Medicine (Proceedings of the 2002 Miami
*Nature Biotechnology* Winter Symposium)
*TheScientificWorld* 2002, 2(S2), 104–105
ISSN 1532-2246; DOI 10.1100/tsw.2002.49

### DETECTION OF MEMBRANE PROTEIN INTERACTIONS USING SPLIT UBIQUITIN-BASED MEMBRANE YEAST TWO-HYBRID TECHNOLOGY

Safia Thaminy[1], Daniel Auerbach[2], Barbara Galeuchet-Schenk[2], Wiep Scheper[3], Karin Römisch[3], and Igor Stagljar[1,2,*]
[1]Institute of Veterinary Biochemistry and Molecular Biology, University of Zurich, Switzerland; [2]Dualsystems Biotech Inc, Zurich, Switzerland; [3]University of Cambridge, CIMR, U.K.
stagljar@vetbio.unizh.ch and stagljar@dualsystems.com

**INTRODUCTION.** Analysis of membrane protein interactions is difficult due to the hydrophobic nature of such proteins, which makes them ill-suited to use in the traditional two-hybrid assay. This is a substantial problem since approximately 40% of all proteins in a typical eukaryotic cell are thought to be anchored in the lipid bilayer[1]. Because of their accessibility, membrane proteins, in particular those spanning the plasma membrane, are also of considerable diagnostic and therapeutic importance. Thus, understanding the physiology of membrane proteins and the way these proteins communicate in the cell is of high biological importance.

**METHODS.** We have recently developed a new genetic method for the *in vivo* detection of membrane protein interactions in *Saccharomyces cerevisiae*[2,3]. The system uses the split-ubiquitin approach first described by Johnsson and Varshavsky[4] based on the observation that covalent addition of ubiquitin polypeptides can recruit particular proteases for the specific degradation of target proteins. In our system, interaction between two membrane proteins results in ubiquitin reconstitution and leads to the proteolytic cleavage and subsequent release of a transcription factor which triggers the activation of a reporter system enabeling easy detection[2,3].

**RESULTS.** Our central goal is to use a membrane-based yeast two-hybrid technology to identify proteins that interact with a given yeast or human membrane target protein fused to Cub-PLV domain. For that purpose, yeast and human cDNA libraries were constructed and fused either N- or C- terminally to the NubG domain. In addition, we are combining a membrane-based yeast two-hybrid approach to the reverse two-hybrid strategy[5] to screen a library of peptides or small molecules in which preventing the interaction between two membrane protein partners provides a selective advantage for yeast. Such an approach should enable identification of dissociating peptides or small molecules that are able to dissociate the interaction between two membrane proteins X and Y.

**DISCUSSION.** All cell processes involve highly regulated protein-protein interactions. Here, we describe the development of a new technology to the study of membrane protein interactions. A major advantage of our membrane-based yeast two-hybrid system is that it can be used to detect not only interactions between two membrane proteins, but also the interaction between membrane and cytoplasmic protein. The ability to monitor the activity and interactions between membrane proteins in their native cellular compartment should prove useful in screening for proteins and drugs that alter their binding properties.

**ACKNOWLEDGMENT.** The I.S. group is supported by grants from the Bonizzi-Theler Stiftung, zürcher Krebsliga, Gebert-Rüf Stiftung, Walter Honegger Stiftung and Swiss National Science Foundation (Nr. 31-58798.99).

**REFERENCES**

1. Goffeau, A. et al. (1996) Science 546, 563–567.
2. Stagljar, I., Korostensky, C., Johnsson, N., and te Heesen, S. (1998) Proc. Natl. Acad. Sci. U. S. A. 95, 5187–5192.
3. Stagljar, I. and te Heesen, S. (2000) Methods Enzymol. 327, 190–198.
4. Johnsson, N. and Varshavsky, A. (1994) Proc. Natl. Acad. Sci. U. S. A. 91, 10340–10344.
5. Vidal, M., Brachmann, R.K., Fattaey, A., Harlow, E., and Boeke, J.D. (1996) Proc. Natl. Acad. Sci. U. S. A. 93, 10315–10320.
